# A Reliable Fracture Angle Determination Algorithm for Extended Puck’s 3D Inter-Fiber Failure Criterion for Unidirectional Composites

**DOI:** 10.3390/ma14216325

**Published:** 2021-10-23

**Authors:** Yaohua Gong, Tao Huang, Xun’an Zhang, Purong Jia, Yongyong Suo, Shuyi Zhao

**Affiliations:** 1 School of Mechanics, Civil Engineering and Architecture, Northwestern Polytechnical University, Xi’an 710129, China; gongyh@mail.nwpu.edu.cn (Y.G.); jiaoping@nwpu.edu.cn (X.Z.); prjia@nwpu.edu.cn (P.J.); yysuo09@mail.nwpu.edu.cn (Y.S.); 2 AECC Commercial Aircraft Engine Co., Ltd., Shanghai 201100, China; zsy_gtlx@163.com

**Keywords:** extended Puck’s criterion, UD composites, fracture angle, stress exposure, algorithm

## Abstract

Determination of the fracture angle and maximum exposure value of extended Puck’s 3D inter-fiber failure (IFF) criterion is of great importance for predicting the failure mechanism of unidirectional fiber-reinforced composites. In this paper, a reliable semi-analytical algorithm (RSAA) is presented for searching fracture angle and corresponding exposure value for the extended Puck’s failure criterion. One hundred million cases are tested for verifying the accuracy of the present and other algorithms on Python using the strength-value-stress-state combinations more universal than those in previous literatures. The reliability of previous algorithms is discussed and counterexamples are provided for illustration. The statistical results show RSAA is adequate for implementation in extended Puck’s criterion and much more reliable than previous algorithms. RSAA can correctly predict the results with a probability of over 99.999%.

## 1. Introduction

Plenty of failure criteria for fiber-reinforced unidirectional (UD) composites have been proposed during the past decades. Among all the failure criteria, the 3D inter-fiber failure (IFF) criterion developed by Puck and Schürmann [1] was ranked high in the “World Wide Failure Exercise I” (WWFE-I) [2] and “World Wide Failure Exercise II” (WWFE-II) [3] due to its good predictions compared with the provided experimental data. Recently, Gu and Chen [4] extended the Puck’s failure criterion, which was expected to be applicable for different types of UD composites. Puck’s failure criterion is developed based on a physical hypothesis that the fracture plane is determined by the shear stresses and normal stress acting on this plane [5]. However, the shear and normal stresses vary on different potential fracture planes and the plane with the highest exposure value is the actual fracture plane. Hence the criterion can not only predict the onset of failure but also can determine the orientation of fracture plane.

For a UD composites with an arbitrary 3D stress state and uncertain material properties, it is more important to know its potential fracture angle before predicting the onset of failure. Due to this reason, Puck [1] and VDI [6] proposed a stepwise search algorithm (SSA) to locate the fracture angle which relied on the degree-to-degree scanning of the potential fracture plane. Within an accuracy of 1°, it will lead to 180 points to be calculated. Higher accuracy leads to more computational effort. Apparently, an efficient and reliable algorithm is required to implement the Puck’s criterion. 

Later, Wiegand et al. [7] proposed an accurate and numerically efficient fracture angle search algorithm called Extended Golden Section Search (EGSS) for Puck’s IFF criterion. EGSS first uses the Golden Section Search (GSS) [8] algorithm to quickly bracket the maximum and then apply a curve interpolation technique called Inverse Parabolic Interpolation (IPI) to locate the maximum. According to Wiegand et al., as few as 6 evaluated points need to be calculated to determine the fracture angle. 

However, Schirmaier et al. [9] have proved that EGSS is only valid for a certain part of the occurring stress states and corresponding stress exposure curves. On certain cases, EGSS will put away the global maximum in the first optimization step and locate the local maximum. Subsequently they proposed a Selective Range Golden Section Search (SRGSS) algorithm. At first, they examined the possible stress exposure curves with different strength-value-stress-state combinations which are performed on MATLAB. The basic idea of SRGSS is to isolate all the maximum-containing ranges and then to localize the maximum using GSS algorithm. According to Schirmaier et al., at most 36 supporting points are required in case of a curve with three maxima. SRGSS is less efficient than EGSS, however, reliability is increased. 

Thomson et al. [10] recently proposed a semi-analytical algorithm (SAA) to determine the fracture angle using a new efficient implementation of Puck’s criterion. One of the main focuses of the literature is to eliminate the additional computational cost of the fracture angle search. Wang et al. [11] proposed Improved Analytical Approximation Golden Section Search (IAAGSS) algorithm, an extension of the algorithm proposed by Thomson et al., which can significantly increase the computation accuracy. However, the present paper works out that the algorithms proposed by Thomson et al. and Wang et al. are not sufficient for predicting the fracture angle and stress exposure value.

So far, several algorithms are available for implementing the Puck’s 3D IFF criterion, however, the accuracy comparison between these algorithms has not been seen yet. The work presented in this paper aims to determine the actual fracture plane in extended Puck’s 3D IFF criterion. The basic principle is to ensure the reliability of the results. On this basis, less computational effort is satisfied compared with SSA. The rest of this paper is organized as follows: Section 2 introduces the extended Puck’s failure criterion. Section 3 and Section 4 discuss the existing methods and the present method for searching the potential fracture plane. Section 5 gives the reliability discussion of the existing algorithms and the comparison results between the algorithms above and the present algorithms. Furthermore, present algorithm is implemented in ABAQUS 2020 and biaxial strength properties are calculated. Concluding remarks are given in Section 6.

## 2. Extension of Puck’s IFF Criterion

### 2.1. Puck’s 3D Inter-Fiber Failure Criterion

**Figure 1:**
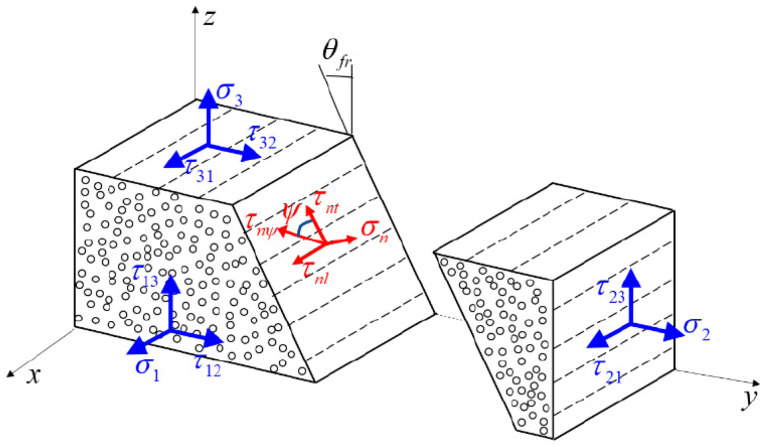
Stress components acting on potential fracture plane.

The Puck’s IFF criterion is based on the Mohr-Coulomb theory and assumes transverse isotropy of the UD composites. Failure will occur on the fracture plane where only shear stresses, $\tau_{nt}$ and $\tau_{nl}$, and normal stress $\sigma_{n}$ exist, which is illustrated in Figure 1. These three stresses can be calculated from $\sigma_{2},\sigma_{3},\tau_{12},\tau_{13},\tau_{23}$ using Equations (1)–(3):

(1) $$\sigma_{n}(\theta )=\sigma_{2}\cos^{2}\theta +\sigma_{3}\sin^{2}\theta +2\tau_{23}\sin\theta \cos\theta$$

(2) $$\tau_{nt}(\theta )=(\sigma_{3}-\sigma_{2})\sin\theta \cos\theta +\tau_{23}(\cos^{2}\theta -\sin^{2}\theta )$$

(3) $$\tau_{nl}(\theta )=\tau_{12}\cos\theta +\tau_{13}\sin\theta$$

Puck’s IFF criterion for unidirectional composites distinguishes the failure mechanisms between $\sigma_{n}\geq 0$ and $\sigma_{n}<0$. For each condition, Puck introduces the stress exposure value $f_{IFF}$, given below, to measure the fracture risk and the fracture occurs when $f_{IFF}$ reaches one. For a given strength-value-stress-state combination, the angle θ is a variable. The problem is thus converted to finding the fracture angle which makes exposure value $f_{IFF}$ maximum.

(4) $$f_{IFF}=\{\begin{array}{cc} \sqrt{{\left(\frac{1}{R_{⊥}^{A+}}-\frac{p_{⊥\psi }^{\left(+\right)}}{R_{⊥\psi }^{A}}\right)}^{2}\sigma_{n}^{2}(\theta )+{\left(\frac{\tau_{nt}(\theta )}{R_{⊥⊥}^{A}}\right)}^{2}+{\left(\frac{\tau_{nl}(\theta )}{R_{⊥∥}}\right)}^{2}}+\frac{p_{⊥\psi }^{\left(+\right)}}{R_{⊥\psi }^{A}}\sigma_{n}(\theta ) & \;\mathrm{for}\;\sigma_{n}\geq 0\\ \sqrt{{\left(\frac{p_{⊥\psi }^{\left(-\right)}}{R_{⊥\psi }^{A}}\sigma_{n}(\theta )\right)}^{2}+{\left(\frac{\tau_{nt}(\theta )}{R_{⊥⊥}^{A}}\right)}^{2}+{\left(\frac{\tau_{nl}(\theta )}{R_{⊥∥}}\right)}^{2}}+\frac{p_{⊥\psi }^{\left(-\right)}}{R_{⊥\psi }^{A}}\sigma_{n}(\theta ) & \;\mathrm{for}\;\sigma_{n}<0 \end{array}$$

with:

(5) $$\frac{p_{⊥\psi }^{\left(+,-\right)}}{R_{⊥\psi }^{A}}=\frac{p_{⊥⊥}^{\left(+,-\right)}}{R_{⊥⊥}^{A}}\cos^{2}\varphi +\frac{p_{⊥∥}^{\left(+,-\right)}}{R_{⊥∥}^{A}}\sin^{2}\varphi$$

(6) $$\cos^{2}\varphi =\frac{\tau_{nt}^{2}(\theta )}{\tau_{nt}^{2}(\theta )+\tau_{nl}^{2}(\theta )}$$

(7) $$R_{⊥⊥}^{A}=\frac{Y_{c}}{2(1+p_{⊥⊥}^{-})}$$

(8) $$R_{⊥∥}^{A}=S_{21}$$

Here, $Y_{c}$ means the transverse compressive strength and $S_{21}$ denotes the longitudinal shear strength of UD composite.

### 2.2. Determination of the Parameters

Puck et al. have already provided inclination parameters [12] for typical GFRP/epoxy (glass fiber reinforced plastic) and CFRP/epoxy (carbon fiber reinforced plastic) UD composites. These materials are regarded as intrinsically brittle materials. However, Gu and Chen [4] recently extended the Puck’s failure criterion and found that the parameters are not adequate for materials with low transverse compression/tension ratios. UD composites are divided into three categories according to the researchers: semi-brittle materials, brittle materials, and intrinsically brittle materials. Some of the parameters used in Puck’s criterion may be different for different types of composite materials. 

There are three strength parameters, $R_{⊥⊥}^{A},R_{⊥∥}^{A},R_{⊥}^{A+}$, and four inclination parameters, $p_{⊥∥}^{\left(+\right)},p_{⊥∥}^{\left(-\right)},p_{⊥⊥}^{\left(+\right)},p_{⊥⊥}^{\left(-\right)}$, in Puck’s IFF criterion. $R_{⊥⊥}^{A},R_{⊥∥}^{A}$ can be calculated using Equations (7) and (8). Inclination parameters $p_{⊥∥}^{\left(+\right)},p_{⊥∥}^{\left(-\right)}$ should be obtained from experiments. In this paper, recommended values are utilized for the two parameters. 

According to Puck et al., $p_{⊥⊥}^{\left(+\right)}=p_{⊥⊥}^{\left(-\right)}=p_{⊥⊥}$ is suggested due to that these two parameters should be approximately of the same magnitude [5]. Up to now, there are two unknown parameters left, $R_{⊥}^{A+},p_{⊥⊥}$, which can be determined using the following equations:

Semi-brittle materials:

(9) $$\{\begin{array}{c} \frac{{\left(a+Y_{T}\right)}^{2}}{4\left(1-b\right)}={\left(R_{⊥⊥}^{A}\right)}^{2}\\ S_{23}^{2}+\frac{a^{2}}{4\left(1-b\right)}={\left(R_{⊥⊥}^{A}\right)}^{2} \end{array}$$

Brittle materials:

(10) $$\{\begin{array}{c} R_{⊥}^{At}=Y_{T}\\ S_{23}^{2}+\frac{a^{2}}{4\left(1-b\right)}={\left(R_{⊥⊥}^{A}\right)}^{2} \end{array}$$

Intrinsically brittle materials:

(11) $$\{\begin{array}{c} R_{⊥}^{A+}=Y_{T}\\ \frac{a}{2\left(1-b\right)}=S_{23} \end{array}$$

with

(12) $$\{\begin{array}{c} a=2p_{⊥⊥}R_{⊥⊥}^{A}\\ b=\frac{{\left(R_{⊥⊥}^{A}\right)}^{2}-2p_{⊥⊥}R_{⊥⊥}^{A}R_{⊥}^{A+}}{{\left(R_{⊥}^{A+}\right)}^{2}} \end{array}$$

$Y_{T}$ means the transverse tensile strength of UD composite. The shear strength $S_{23}$ is difficult to obtain experimentally because it is hard to induce a state of uniform shear and eliminate the geometry effect of specimens [13,14]. Thus, a simple formula provided by Christenson [15] is used to determine $S_{23}$ as follows:

(13) $$S_{23}=\min\left(\sqrt{\frac{1+Y_{T}/Y_{C}}{3+5Y_{T}/Y_{C}}Y_{T}Y_{C}},Y_{T}\right)$$

## 3. Existing Algorithms for Searching the Fracture Angle

The search of fracture angle represents an optimization problem. What we should do is to find the angle which makes the stress exposure in Equation (4) greatest in given strength-value-stress-state case. However, purely analytical solutions are not available due to the complexity of the formulas, and therefore, semi-analytical and numerical approaches are applied.

### 3.1. SRGSS Algorithm Proposed by Schirmaier et al.

Schirmaier et al. proposed a fast and reliable fracture angle search algorithm called Selective Range Golden Section Search. Before SRGSS, the authors have conducted a research to examine possible stress exposure curves using MATLAB. At first, strength values are permuted between the ranges ($Y_{T}$: 25~65, $Y_{C}$: 120~220, $S_{12}$: 70~90). Then the stress values are permuted between the relating strength values. Therefore, the stress exposure curves can be obtained. According to Schirmaier et al. all the curves have the following characteristics in common:

1. The curves are smooth;
2. The curves have at most three local maxima;
3. The distance between two adjacent local maxima is larger than 25°.

Based on the above three characteristics, SRGSS is developed, the main idea of which is to isolate all the local maxima and then localize the maximum in the maximum-containing range using GSS algorithm. At first, the whole range (−90°~90°) is evenly divided into 18 parts. Owing to that the minimum distance is larger than 25°, two adjacent maxima cannot be distributed at the neighboring parts. Thus, the maximum-containing range can be found when a supporting point has two smaller neighbors. Then the global maximum can be determined by comparing the previous two or three local maxima.

### 3.2. Semi-Analytical Algorithm Proposed by Thomson et al.

Thomson et al. proposed a semi-analytical solution to the puck’s IFF criteria by using an approximation of the potential fracture plane, and in this way, the computational effort of the search is greatly reduced.

First, the expressions of three tractions in Equations (1)–(3) are simplified into a single cosine function using basic trigonometric relations. In this way, the location, namely potential angle listed in Table 1, of its maximum for each traction can be easily determined. The Puck’s IFF criterion is a combination of these tractions and the authors believe these angles can be used to approximate the global maximum due to that the fracture angle will tend to the maximum location associated with one of the three tractions when the traction is dominant with respect to the other two tractions. In practice, the simplest approach described in [10] is to consider the potential angles as initial candidates for the resulting global maximum and take the plane with the highest exposure as starting point for a numerical search of EGSS proposed by Wiegand et al. [7]. According to Thomson, this search algorithm can significantly reduce the computational effort and the result can converge on the global maximum instead of the local ones. 

### 3.3. IAAGSS Algorithm Proposed by Wang and Zhao

The search algorithm proposed by Wang and Zhao is an extension of the algorithm proposed by Thomson et al. [10]. They take six angles, listed in Table 1, as initial candidates based on the hypothesis that when one of ${\left|\sigma_{n}\right|}^{\max},\;{\left|\tau_{nt}\right|}^{\max},\;{\left|\tau_{nl}\right|}^{\max}$ takes the dominant position with respect to the others, the resulting fracture plane will tend to the angles mentioned above. The difference between the two algorithms is that Wang takes both maximum and minimum values of the three tractions into consideration. The next step is to search the global maximum based on the six angles. In Wang’s previous literature [16], an Analytical Approximation Golden Section Search (AAGSS) algorithm is proposed. AAGSS takes the highest two exposures (from the six initial candidates) as starting points for GSS algorithm. While IAAGSS improves the AAGSS algorithm and it takes the two planes neighboring to the plane with the highest exposure as starting points for GSS algorithm. The authors also conclude that IAAGSS can converge on the exact global maximum. In this paper, only IAAGSS is utilized for comparison with the present algorithm.

## 4. Proposed Changes for Searching the Fracture Angle

As mentioned in the introduction, the main purpose of this paper is to improve reliability of the algorithm for determining the fracture angle and exposure value of extended Puck’s 3D IFF criterion. The algorithm proposed in this paper is based on that proposed by Thomson et al. [10]. The basic idea of this algorithm is using a semi-analytical approximation to localize all the local maxima near the global maximum and then using the GSS to determine the exact global maximum.

As discussed in Section 3.2, the three tractions in Equations (1)–(3) have to be simplified into single cosines using the trigonometric relations listed below:

(14) $$\sin\theta \cos\theta =\frac{1}{2}\sin2\theta$$

(15) $$\cos^{2}\theta =\frac{1+\cos2\theta }{2},\sin^{2}\theta =\frac{1-\cos2\theta }{2}$$

(16) $$a\cos\theta +b\sin\theta =\sqrt{a^{2}+b^{2}}\cos\left(\theta -\arctan\left(\frac{b}{a}\right)\right)$$

In this way, the expressions of the three tractions are given below:

(17) $$\begin{array}{l} \sigma_{n}=\frac{1}{2}\left(\sigma_{22}+\sigma_{33}\right)+\sqrt{\frac{1}{4}{\left(\sigma_{22}-\sigma_{33}\right)}^{2}+\tau_{23}^{2}}\cos\left(2\theta -\arctan\left(\frac{2\tau_{23}}{\sigma_{22}-\sigma_{33}}\right)\right)\\ \tau_{nt}=\sqrt{\frac{1}{4}{\left(\sigma_{22}-\sigma_{33}\right)}^{2}+\tau_{23}^{2}}\cos\left(2\theta +\arctan\left(\frac{\sigma_{22}-\sigma_{33}}{2\tau_{23}}\right)\right)\\ \tau_{nl}=\sqrt{\tau_{12}^{2}+\tau_{13}^{2}}\cos\left(\theta -\arctan\left(\frac{\tau_{13}}{\tau_{12}}\right)\right) \end{array}$$

It should be noted that the expressions of $\tau_{nt},\tau_{nl}$ are not the same with those provided in Thomson et al. [10] and Wang et al. [11,16]. Most probably, the trigonometric formulas are used by the authors and there are misprints in their papers. After obtaining the simplified expressions of tractions, the locations of maximum and minimum can easily be defined, listed as $\theta_{1}\sim \theta_{5}$ in Table 2. Some explanations should be stated here that the frequency of $\tau_{nl}$ is 180°, which is half of that of $\sigma_{n}$ and $\tau_{nt}$. While the fracture plane is searched within the interval (−90°, 90°). Hence, only one potential angle, $\theta_{5}$, for $\tau_{nl}$ is obtained. For $\sigma_{n}$ and $\tau_{nt}$, both the maximum and minimum locations are calculated. In this way, the exposure curves are split in order to prevent the simultaneous occurrence of local maximum and minimum values in the same interval, which may affect the following GSS algorithm for search local maximum. It should be noticed that all these potential angles should be in the range of (−90°, 90°), so the angles are calculated using different formulas according to different situations. 

However, in actual cases, the final results are related to the interaction of the three tractions and the global maximum lies in between the angles for almost all the state cases. Hence, several initial candidates, $\theta_{6}\sim \theta_{10}$, should be added in consideration of the stress weights. An operator, {}, is defined here to calculate the new added angles. An illustrative example for $\left\{\theta_{1},\theta_{3}\right\}$ is shown in Equation (18). After the operator calculation, the newly added angle should be transferred to range (−90°, 90°) according to the frequency of 180° if it is out of range.

(18) $$\left\{\theta_{1},\theta_{3}\right\}=\{\begin{array}{c} \left(\theta_{1}\left|\sigma_{n}^{\max}\left(\left|1/R_{⊥}^{At}-p\right|+p\right)\right|+\theta_{3}\tau_{nt}^{\max}/R_{⊥⊥}^{A}\right)/\left(\left|\sigma_{n}^{\max}\left(\left|1/R_{⊥}^{At}-p\right|+p\right)\right|+\tau_{nt}^{\max}/R_{⊥⊥}^{A}\right)\;\mathrm{for}\;\left|\theta_{1}-\theta_{3}\right|\leq 90{}^{\circ}\\ \left(\left(\theta_{1}+180{}^{\circ}\right)\left|\sigma_{n}^{\max}\left(\left|1/R_{⊥}^{At}-p\right|+p\right)\right|+\theta_{3}\tau_{nt}^{\max}/R_{⊥⊥}^{A}\right)/\left(\left|\sigma_{n}^{\max}\left(\left|1/R_{⊥}^{At}-p\right|+p\right)\right|+\tau_{nt}^{\max}/R_{⊥⊥}^{A}\right)\;\mathrm{for}\;\left|\theta_{1}-\theta_{3}\right|>90{}^{\circ}\;\mathrm{and}\;\theta_{1}<\theta_{3}\\ \left(\theta_{1}\left|\sigma_{n}^{\max}\left(\left|1/R_{⊥}^{At}-p\right|+p\right)\right|+\left(\theta_{3}+180{}^{\circ}\right)\tau_{nt}^{\max}/R_{⊥⊥}^{A}\right)/\left(\left|\sigma_{n}^{\max}\left(\left|1/R_{⊥}^{At}-p\right|+p\right)\right|+\tau_{nt}^{\max}/R_{⊥⊥}^{A}\right)\;\mathrm{for}\;\left|\theta_{1}-\theta_{3}\right|>90{}^{\circ}\;\mathrm{and}\;\theta_{1}>\theta_{3} \end{array}$$

Here:

(19) $$p=\max\left(p_{⊥⊥}/R_{⊥⊥}^{A},p_{⊥∥}/R_{⊥∥}^{A}\right)$$

**Figure 2:**
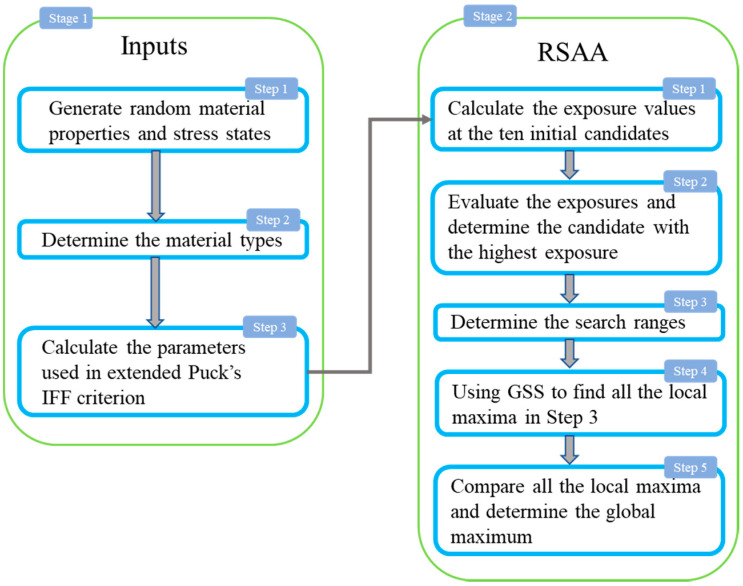
Flowchart of present algorithm.

**Figure 3:**
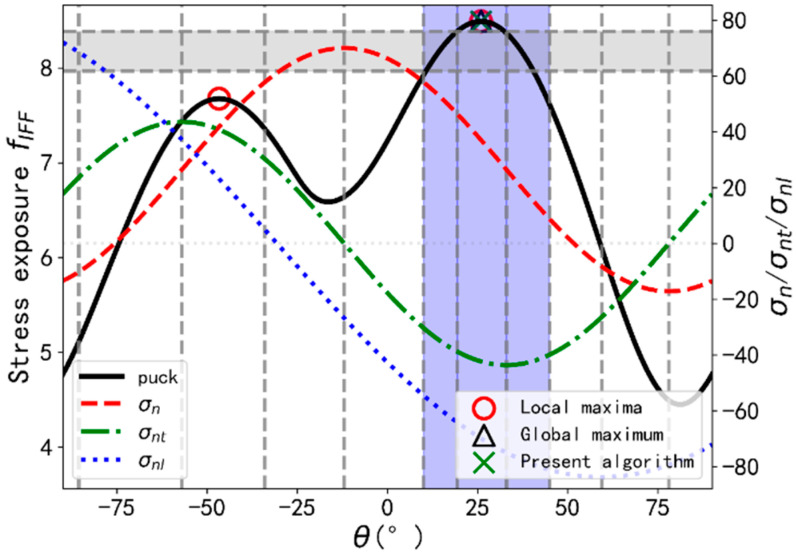
Illustration example for present algorithm with the curves for three tractions.

In order to test the reliability of the fracture angle search algorithm, Python scripts are used to implement all the algorithms due to its outstanding performance in numerical calculation. The process of RSAA for extended Puck’s IFF criterion is shown in Figure 2. Inputs should be generated randomly in stage-1 for the material properties and stress states, ranges of which are shown in Table 3. The types of composites include the semi-brittle materials with $Y_{C}/Y_{T}\in \left(1,\;2.5\right)$, brittle materials with $Y_{C}/Y_{T}\in \left(2.5,\;3.45\right)$ and intrinsically brittle materials with $Y_{C}/Y_{T}>3.45$ according to Gu and Chen [4]. Each of stress components are set to be in the range of [−100, 100] MPa which can represent all the possible stress state combinations. All the strength and stress parameters are randomly selected from the given ranges. It should be mentioned that the value of stress component may be greater than the corresponding strength and thus may lead to the stress exposure $f_{IFF}$ greater than one. However, $f_{IFF}$ and the stress component combination are linearly positively correlated, thus the location with the highest exposure, namely the fracture plane, remains the same. In step 3, the related parameters required in extended Puck’s IFF criterion are determined based on the material types using equations in Section 2.2. In stage 2, RSAA is implemented to find the angle that makes the exposure value $f_{IFF}$ highest in the range between −90 and 90 degrees. In the first two steps, the exposure values of the ten initial supporting points, $\theta_{1}\sim \theta_{10}$, are calculated, and then select the highest exposure, the value of which is assumed to be $f_{0}$. In step 3, to localize the potential angle ranges that contain the global maximum, the supporting points whose exposures greater than $0.96f_{0}$ are recorded. In step 4, each supporting point, recorded in step 3, together with the two neighboring points form two search ranges. Then, all local maxima can be found using GSS algorithm. At last, global maximum and corresponding fracture angle are determined by comparing the aforementioned local maxima. An illustration example for present algorithm is shown in Figure 3. Exposure value greater than $0.96f_{0}$ is depicted in the gray area. Ranges selected for GSS algorithm are depicted in light blue area.

Monte Carlo simulations were implemented in Python to obtain the characteristics due to the complexity of the Puck’s criterion and stress state. To verify the accuracy of the present algorithm, outcomes obtained from SRGSS, SAA, and IAAGSS are compared with the outcomes obtained from Puck’s original algorithm (SSA) with an accuracy of 0.01° regardless of the search efficiency under the same strength-stress combinations. A total of 100,000,000 combinations were tested. 

## 5. Results and Discussions

### 5.1. Reliability of the Existing Algorithms

#### 5.1.1. SRGSS Algorithm

For SRGSS algorithm, its main prerequisites are those that the stress exposure curves have at most three local maxima and the minimum distance between two adjacent maxima is no less than 25°. Besides, the method of localizing the local maxima is to compare the supporting points and obtain the points with two smaller neighbors. However, after thirty million random tests, the results show that the curves may have up to four local maxima and the minimum distance can be lower than 25° (minimum distance is about 3° based on our results). Details of the numbers of local maxima are listed in Table 4.

**Figure 4:**
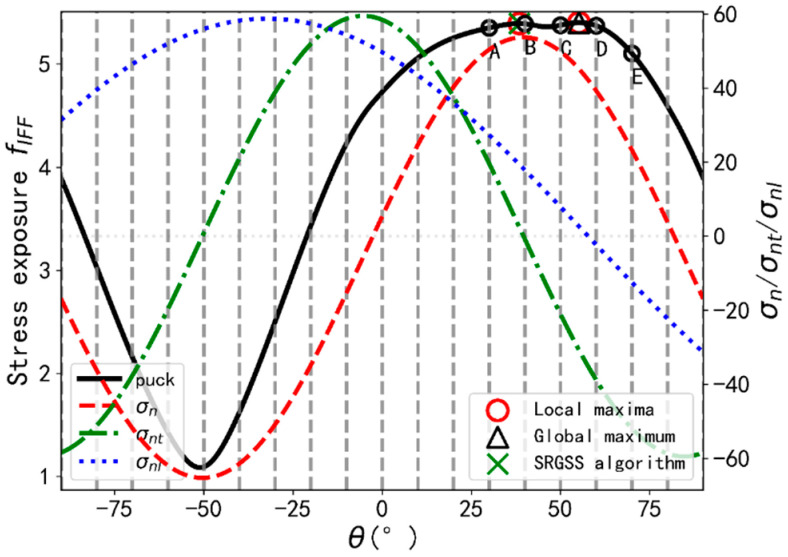
Illustration example for SRGSS algorithm.

Figure 4 provides an illustration example that the SRGSS finally locates the local maximum instead of the global maximum. The stress exposure values f at supporting points A, B, C, D, and E are 5.3519, 5.3918, 5.3778, 5.3727, and 5.1053 respectively. It is obvious that $f_{C}>f_{D}>f_{E}$. Hence, no local maximum exists in range (C, E) according to SRGSS. Therefore, the search range is (A, C) and it results in the local maximum using GSS algorithm. Actually, exposure values obtained from SSA and SRGSS algorithms are 5.4027 and 5.3941 respectively. Besides, there is another aspect which may also not be able to determine the global maximum. Since the minimum distance between two adjacent supporting points could be smaller than the conclusion from Schirmaier et al. [9], two local maxima may exist in one search range and the same problem may arise using GSS as is discussed by Schirmaier et al. 

#### 5.1.2. Semi-Analytical Algorithm Proposed by Thomson et al.

**Figure 5:**
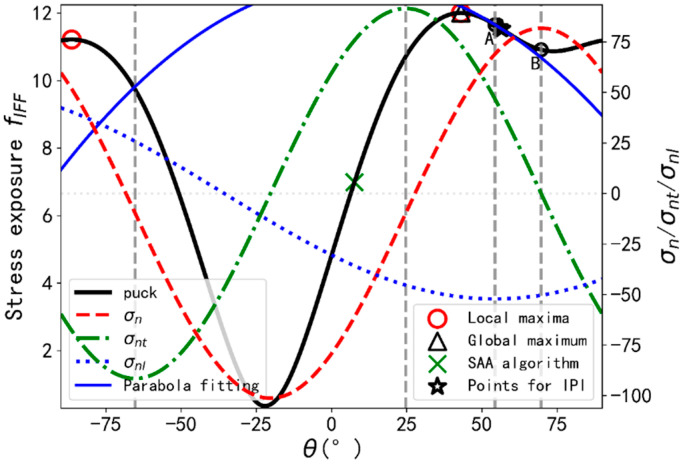
Illustration example for SAA algorithm.

The SAA may locate the incorrect global maximum, even the stress exposure curve has only one local maximum and an illustration example is shown in Figure 5. It should be stated that in the definition the $\theta_{1}$ may locate the minimum of $\sigma_{n}$ in certain cases and we have used the form of $\theta_{1}$ proposed in this paper, hence it can exactly locate the maximum of $\sigma_{n}$ in all stress states. Other initial candidates such as $\theta_{4}$ are also modified based on our results. Another point that should also be mentioned here is that according to Thomson et al., the four initial candidates are calculated and take the plane with the highest exposure as the starting point for a numerical search of EGSS described in [7]. However, an ending point is also required to implement the EGSS algorithm. Hence, we take the planes with the highest two exposures as starting and ending intervals for EGSS, the method of which is also conducted in literature [17], which has used the algorithm proposed by Thomson et al. [10].

As is observed from the figure, the real global maximum is not in the range (A, B), the highest two exposures. The EGSS algorithm is then implemented and after several times GSS, the three points are determined for IPI, which results in the incorrect global maximum. 

#### 5.1.3. IAAGSS Algorithm

**Figure 6:**
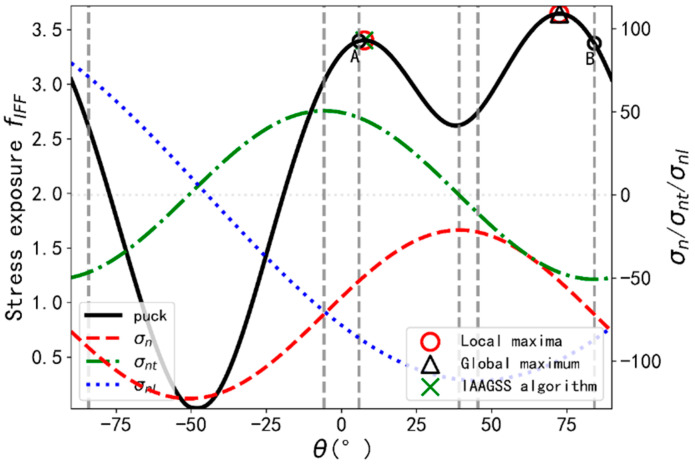
Illustration example for IAAGSS algorithm.

The IAAGSS algorithm is an extension of SAA and used the initial candidates related to the three tractions. Similarly, the initial angles may not be capable to locate the maximum or minimum of the tractions or may be outside the range of (−90°, 90°). So, the modifications have been made so as to achieve what the authors expect. An illustration example is depicted in Figure 6. The stress exposure values at point A and B are 3.3958 and 3.3756 respectively. According to IAAGSS, candidate at point A is with the highest exposure and the range used for GSS is depicted in blue region in Figure 6. Obviously, a local maximum is obtained rather than a global one.

### 5.2. Comparison between the Present and Existing Algorithms

In this part, the final results, including the fracture angle and stress exposure value, are compared using present and the existing algorithms. The referenced results, namely the real fracture angle and exposure value, are determined using SSA. Thus, the distance ($\theta_{d}$) between real fracture angle ($\theta_{r}$) and the angles obtained from different algorithms ($\theta_{a}$) and the relative error ($r_{f}$) between the real exposure value ($f_{r}$) and exposures obtained using different algorithms ($f_{a}$) are defined as:

(20) $$\theta_{d}=\{\begin{array}{c} \left|\theta_{a}-\theta_{r}\right|\;\;\;\;\mathrm{if}\;\left|\theta_{a}-\theta_{r}\right|\leq 90^{{}^{\circ}}\\ 180^{{}^{\circ}}-\left|\theta_{a}-\theta_{r}\right|\;\;\mathrm{if}\;\left|\theta_{a}-\theta_{r}\right|>90^{{}^{\circ}} \end{array}$$

(21) $$r_{f}=\frac{\left|f_{a}-f_{r}\right|}{f_{r}}$$

The angle distance and the relative error of exposure value obtained from the SSA and other algorithms were compared. Considering that the results from the SSA may not be the exact global maximum due to the incremental step of 0.01°, angle distances larger than 1° and relative errors of exposure value larger than 0.01% are shown in Table 5 and Table 6. The angle distance and relative error are classified into four separate groups. It should be highlighted that the RSAA has obvious advantages over other algorithms in predicting both the fracture angle and related exposure value. The incorrect rates of the RSAA for angle distance and exposure relative error are only 5.7% and 1.74%, respectively, of those of SRGSS. Both the RSAA and IAAGSS are extended from the SAA, but the RSAA can correctly predict the results with a probability of over 99.999%.

It can be seen from Table 5 that there is still a small probability that the RSAA will miss the accurate fracture angle with angle distances greater than 1°. Nonetheless, the relative error of stress exposure value shows no case greater than 1% for the RSAA (actually, the maximum is 0.27%). This phenomenon is attributed to the fact that the stress exposure curve may have two or more very close local maxima in certain cases even though the corresponding angles may vary obviously (e.g., in the uniaxial compression stress state, the exposure curve has two identical local maxima, but the corresponding fracture angles are 53° and −53°).

The efficiency of all the algorithms including the Puck’s original algorithm (SSA) under same strength-value-stress-state combinations with accuracy of 0.1° is compared. The cost time for ten thousand random tests is listed in Table 7. It can be seen the cost time of present algorithm is only about 11% of cost time of SSA. However, the calculation effort is larger than those for SRGSS, SAA, and IAAGSS, but present algorithm has the essential advantage of giving reliable outcomes. In general, RSAA is adequate for predicting the fracture angles and exposures for the extended Puck’s 3D IFF criterion and it is a balance of reliability and efficiency.

### 5.3. Application of Present Algorithm in FE Analysis

**Figure 7:**
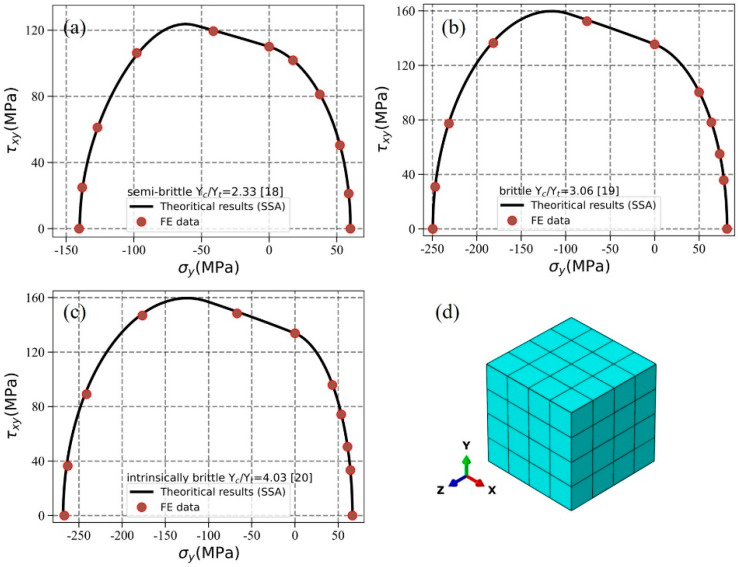
Failure envelopes of different types of UD composites. (**a**) Semi-brittle material; (**b**) brittle material; (**c**) intrinsically brittle material; (**d**) FE model of UD composites.

The present algorithm is implemented in the FE analysis using the material subroutine UMAT in ABAQUS standard. Considering the predicted strengths of multidirectional laminates depend much on the degradation model, UD composites are utilized in this part. A cubic FE model is established to represent the representative volume model of UD composites and x-dir denotes the longitudinal direction, which is shown in Figure 7d. Different types of UD composite materials including semi-brittle [18], brittle [19], and intrinsically brittle [20] materials are considered. Biaxial strength properties in $\sigma_{y}-\tau_{xy}$ stress space are obtained by applying periodical boundary conditions to FE model. Details for RVE model, periodical boundary conditions, and loadings can refer to our previous literature [21]. Figure 7a–c indicates that the FE results using present algorithm correlate well with the theoretical results using SSA algorithm with accuracy of 0.1°.

It should be mentioned that the FE results using present algorithm and SRGSS algorithm are the same for the above simulation. The reason of this phenomenon can be attributed to that the stress states of the FE model are very simple, but the stress states can be significantly complicated and differences may appear between different algorithms for laminate and fabric composite material. If the damage location and the related criteria value cannot be accurately determined, it will greatly affect the prediction of strength property and the subsequent damage evolution process. However, as is discussed in Section 5.2, these stress states exist and the present algorithm shows superiority than other algorithms. It can be concluded that using the present algorithm can obtain the accurate results with higher probability compared with others.

## 6. Conclusions

In the present work, the extended Puck’s 3D IFF criterion is utilized for verifying the reliability of the present algorithms for determining the fracture angle and maximum stress exposure value. The algorithm in this paper is based on the SAA proposed by Thomson et al. and the basic idea is to use semi-analytical approximation to localize the local maxima near global maximum and then obtain the global maximum using GSS. Thus, ten initial candidates are required for the present algorithm. 

The reliability and shortcomings of the existing algorithms are discussed and counterexamples of their algorithms are plotted for illustration. Present algorithm avoids these shortcomings. One hundred million strength-stress combination cases were tested to verify the reliability of the existing algorithms. Statistical results indicate that the RSAA is much more reliable than SRGSS, the SAA, and IAAGSS, which make it a practical alternative for the application of Puck’s 3D failure criterion in composite analysis. The computational effort is larger than the other algorithms, however, it is only about one-tenth the time cost of the SSA. Present algorithm is implemented in ABAQUS and biaxial strength properties in $\sigma_{y}-\tau_{xy}$ stress space are calculated, which are in good agreement with theoretical results using SSA. In general, present algorithm is a balance of reliability and efficiency and adequate for evaluating the Puck’s 3D IFF criterion.

## Figures and Tables

**Table 1:** Initial search angles for SAA, IAAGSS.

	Maxima Candidates Location	
	IAAGSS	SAA
$\sigma_{n}$	$\begin{array}{l} \theta_{1}=\frac{1}{2}\arctan\left(\frac{2\tau_{23}}{\sigma_{22}-\sigma_{33}}\right)\;\mathrm{for}\;\left(\sigma_{22}+\sigma_{33}\right)>0\\ \mathrm{or}\;\theta_{1}=\frac{1}{2}\arctan\left(\frac{2\tau_{23}}{\sigma_{22}-\sigma_{33}}\right)+90{}^{\circ}\;\mathrm{for}\;\frac{\tau_{23}}{\sigma_{22}-\sigma_{33}}<0\\ \theta_{1}=\frac{1}{2}\arctan\left(\frac{2\tau_{23}}{\sigma_{22}-\sigma_{33}}\right)-90{}^{\circ}\;\mathrm{for}\;\frac{\tau_{23}}{\sigma_{22}-\sigma_{33}}\geq 0 \end{array}$	$\theta_{1}=\frac{1}{2}\arctan\left(\frac{2\tau_{23}}{\sigma_{22}-\sigma_{33}}\right)$
$\tau_{nt}$	$\theta_{3}=\frac{1}{2}\arctan\left(\frac{\sigma_{22}-\sigma_{33}}{2\tau_{23}}\right)$ $\theta_{4}=-\frac{1}{2}\arctan\left(\frac{\sigma_{22}-\sigma_{33}}{2\tau_{23}}\right)$ $\begin{array}{l} \theta_{5}=90{}^{\circ}+\frac{1}{2}\arctan\left(\frac{\sigma_{22}-\sigma_{33}}{2\tau_{23}}\right)\;\mathrm{for}\;\frac{\sigma_{22}-\sigma_{33}}{2\tau_{23}}<0\\ \mathrm{or}\;\theta_{5}=-90{}^{\circ}+\frac{1}{2}\arctan\left(\frac{\sigma_{22}-\sigma_{33}}{2\tau_{23}}\right) \end{array}$ $\begin{array}{l} \theta_{6}=90{}^{\circ}-\frac{1}{2}\arctan\left(\frac{\sigma_{22}-\sigma_{33}}{2\tau_{23}}\right)\;\mathrm{for}\;\frac{\sigma_{22}-\sigma_{33}}{2\tau_{23}}<0\\ \mathrm{or}\;\theta_{6}=-90{}^{\circ}-\frac{1}{2}\arctan\left(\frac{\sigma_{22}-\sigma_{33}}{2\tau_{23}}\right) \end{array}$	$\theta_{2}=\theta_{1}+45{}^{\circ}$ $\theta_{3}=\theta_{1}-45{}^{\circ}$
$\tau_{nl}$	$\theta_{2}=-\arctan\left(\frac{\tau_{13}}{\tau_{12}}\right)$	$\theta_{4}=-\arctan\left(\frac{\tau_{13}}{\tau_{12}}\right)$

**Table 2:** Initial search angles for present algorithm.

Extreme Angles	Location	Newly Added Angles	Location
$\theta_{1}$	$\begin{array}{l} \frac{1}{2}\arctan\left(\frac{2\tau_{23}}{\sigma_{22}-\sigma_{33}}\right)\;\mathrm{for}\;\left(\sigma_{22}-\sigma_{33}\right)>0\\ \mathrm{or}\;\frac{1}{2}\arctan\left(\frac{2\tau_{23}}{\sigma_{22}-\sigma_{33}}\right)+90{}^{\circ}\;\mathrm{for}\;\tau_{23}>0\\ \frac{1}{2}\arctan\left(\frac{2\tau_{23}}{\sigma_{22}-\sigma_{33}}\right)-90{}^{\circ}\;\mathrm{for}\;\tau_{23}\leq 0 \end{array}$	$\theta_{6}$	$\left\{\theta_{3},\theta_{5}\right\}$
$\theta_{2}$	$\begin{array}{l} \theta_{1}+90{}^{\circ}\;\mathrm{for}\;\theta_{1}<0{}^{\circ}\\ \theta_{1}-90{}^{\circ}\;\mathrm{for}\;\theta_{1}\geq 0{}^{\circ} \end{array}$	$\theta_{7}$	$\left\{\theta_{4},\theta_{5}\right\}$
$\theta_{3}$	$\begin{array}{l} \frac{1}{2}\arctan\left(\frac{\sigma_{22}-\sigma_{33}}{2\tau_{23}}\right)\;\mathrm{for}\;\tau_{23}<0\\ \mathrm{or}\;\frac{1}{2}\arctan\left(\frac{\sigma_{22}-\sigma_{33}}{2\tau_{23}}\right)+90{}^{\circ}\;\mathrm{for}\;\left(\sigma_{22}-\sigma_{33}\right)<0\\ \frac{1}{2}\arctan\left(\frac{\sigma_{22}-\sigma_{33}}{2\tau_{23}}\right)-90{}^{\circ}\;\mathrm{for}\;\left(\sigma_{22}-\sigma_{33}\right)\geq 0 \end{array}$	$\theta_{8}$	$\left\{\theta_{1},\theta_{3}\right\}\;$
$\theta_{4}$	$\begin{array}{l} \theta_{3}+90{}^{\circ}\;\mathrm{for}\;\theta_{3}<0{}^{\circ}\\ \theta_{3}-90{}^{\circ}\;\mathrm{for}\;\theta_{3}\geq 0{}^{\circ} \end{array}$	$\theta_{9}$	$\left\{\theta_{1},\theta_{4}\right\}$
$\theta_{5}$	$\arctan\left(\frac{\tau_{13}}{\tau_{12}}\right)$	$\theta_{10}$	$\left\{\theta_{1},\theta_{5}\right\}$

**Table 3:** Strength ranges and stress ranges for random strength-value-stress-state combinations.

Material Properties	Range	Stress Components	Range
$Y_{T}$	10	$\sigma_{22}$	[−100, 100]
$Y_{C}$	$\left[Y_{T},5Y_{T}\right]$	$\sigma_{33}$	[−100, 100]
$S_{12}$	$\left[0.5Y_{T},4Y_{T}\right]$	$\tau_{12}$	[−100, 100]
$p_{⊥∥}$	[0.15, 0.35]	$\tau_{23}$	[−100, 100]
		$\tau_{13}$	[−100, 100]

**Table 4:** Number of the local maxima and their number of occurrences after twenty-seven million tests.

Number of Tests	Number (*n*) of Local Maxima			
	*n* = 1	*n* = 2	*n* = 3	*n* = 4
30,000,000	12,395,304	17,180,133	420,194	4369

**Table 5:** Frequency of occurrence: angle distance $\theta_{d}$ (°).

Angle Distance (°)	1–5	5–10	10–20	>20
RSAA	1346	568	207	1742
SRGSS	8584	16,339	29,301	13,609
SAA	5,481,459	541,710	486,182	8,409,864
IAAGSS	9025	19,874	106,336	7,002,295

**Table 6:** Frequency of occurrence: relative error of exposure value $r_{f}$ (%).

Relative Error (%)	0.01–0.1	0.1–1	1–10	>10
RSAA	471	132	0	0
SRGSS	29,884	5884	251	21
SAA	7,286,700	6,945,001	3,743,293	496,285
IAAGSS	1,105,654	3,671,127	2,100,743	571

**Table 7:** The cost of calculation of different algorithms.

Algorithms	SSA	SRGSS	SAA	IAAGSS	RSAA
Total time (s)	151.81	7.56	1.25	3.97	17.05
Relative time (%)	100	4.98	0.82	2.62	11.23

## Data Availability

Data is contained within the article.
